# Classification of ginseng berry (Panax ginseng C.A. MEYER) extract using ^1^H NMR spectroscopy and its inhibition of lipid accumulation in 3 T3-L1 cells

**DOI:** 10.1186/1472-6882-14-455

**Published:** 2014-11-24

**Authors:** Seung Ok Yang, Hae Ran Park, Eun Suk Sohn, Sang Won Lee, Hyung Don Kim, Young Chang Kim, Kee Hong Kim, Sae Won Na, Hyung-Kyoon Choi, Mariadhas Valan Arasu, Young Ock Kim

**Affiliations:** Dapartment of food science and engineering, Ewha Womans University, 11-1 Daehyun-dong, Seodaemun-gu, Seoul, 120-750 Republic of Korea; Department of Medicinal Crop Research NIHHS RDA Eumsung, Chungbuk, 369-873 Republic of Korea; Department of Applied Biochemistry, College of Biomedical & Health Science, Konkuk University, Chungju, 380-701 South Korea; College of Veterinary Medicine, ChonBuk National University, Jeonju-si, South Korea; College of pharmacy, Chung-Ang University, Seoul, 156-756 Republic of Korea; Department of Botany and Microbiology, Addiriyah Chair for Environmental Studies, College of Science, King Saud University, P. O. Box 2455, Riyadh, 11451 Saudi Arabia

**Keywords:** Panax ginseng berry, Obesity, Metabolite profiling, Maturation

## Abstract

**Background:**

Panax ginseng is a famous traditional medicine in Korea for its beneficial effect on obesity, cardiac and liver associated diseases. The aim of this study was to investigate the metabolite in Panax ginseng (P. ginseng, Aralicaceae) berries depending on the ripen stages and evaluate its potential inhibition on adipocyte differentiation in 3 T3-L1 cells.

**Methods:**

Different ripening stage samples of P. ginseng berry were analyzed through global metabolite profiling by NMR spectroscopy. Lipid accumulation in the cells was analyzed by Oil Red O staining.

**Results:**

The PLS-DA clearly distinguished P. ginseng berry extract (PGBE) according to the partial ripe (PR), ripe(R) and fully ripe (FR) stage. Lipid accumulation of PGBE was examined by measuring triglyceride content and Oil-Red O staining. These results suggested that the FR stage of PGBE decrease in lipid accumulation during adipocyte differentiation and the amount of threonine, asparagine, fumarate, tyraine, tyrosine, and phenylalanine increased with longer ripening of ginseng berries.

**Conclusion:**

Metabolite profiling of P. ginseng was identified by ^1^H NMR spectra. P. ginseng extract efficiently inhibits adipogenesis in 3 T3-L1 adipocytes concluded that the P. ginseng has the antiobesity properties.

## Background

Obesity is a risk factor for major metabolic disease including type 2 diabetes, atherosclerosis, hyperlipidemia and hypertension and multifactorial syndrome in human [1, 2]. It is an abnormal condition in which the lipids are accumulated in adipose tissues and various kinds of adipokine [3]. Panax ginseng is a perennial plant that has been used as a tonic and for the treatment of various diseases [4–8]. P. ginseng root is normally harvested between the fourth and sixth year of growth. The multiple active constituents such as ginsenosides, polysaccharides, peptides, polyacetylenic alcohols and fatty acids are identified in the P. ginseng root [9]. On the other hand, P. ginseng berry easily be harvested several times after the third year of growth [10]. Quan et al. [11] reported that ginsenoside Re (groups, namely protopanaxatriol-type saponin from ginseng) lowers blood glucose and lipid in high-fat diet fed mice. However, effects on adipocyte differentiation in 3 T3-L1 cells on PGBE have not yet been reported. The chemical composition and biological activities of the P. ginseng berry may differ according to the maturation stage. We have looked for lipid accumulation in adipocyte inhibitory plants using PGBE as an in vitro assay system. During the course of screening, the water extract of P. ginseng berry was significantly inhibited this activity. The metabolic profiling can be useful for quantifying a group of related compounds. There are few previous studies about profiling metabolic compounds of ginseng by using NMR [12, 13]. However, no study reported in differences of the metabolic compounds among different maturation stages in ginseng fruits. The aim of this study was to classify the ginseng berry (Panax ginseng) extract using ^1^H NMR spectroscopy and evaluates its inhibition of lipid accumulation in 3 T3-L1 cells.

## Methods

### Plant material

Three steps berries of five year-old P. ginseng were obtained from a local farm in Eumseong province (GPS N 36° 56’ 34”, E 127° 45’ 14”), Republic of Korea. The collected plants were identified by the botanist in Ginseng Research Institiute, Daegu, South Korea. The collected samples were stored in the Medicinal Crop Research Institute, NIHHS, RDA, with voucher number MCRI-241. Three periods were June 8th, 2012 (12ea), June 18th, 2012 (10ea), and July 16th, 2012 (8ea), respectively. The collected P. ginsengs were classified into three major categories according to their stage of maturation: ripe (R), and fully ripe (FR). The PGBE was freeze-dried and then stored at -70°C before analysis. Voucher specimens were deposited at the Department of Medicinal Crop Research, Rural Development Administration in Republic of Korea (RDAPGBE 201201–201230).

### Sample preparation for ^1^H NMR

PGBE were extracted by adding 1 mL of 100% D_2_O to 30 mg of powdered P. ginseng berries in a micro tube, vortexed for 1 min, and sonicated for 5 min. The materials were then centrifuged at 14000 × rpm for 10 min. KH_2_PO_4_ was added as a buffering agent to 100 mL of D_2_O containing 0.05% 3-(trimethylsilyl)-propionic-2,2,3,3-d4 acid, sodium salt (TSP) as an internal standard for D_2_O. The pH of the D2O used for NMR measurements was adjusted to 6.0 by careful addition of 1 N NaOD and then transferred to a 5 mm NMR tube.

### Data reduction and processing

MestReNova (version 6.0.4) was used to obtain the NMR spectra, which were all automatically binning using Chenomx (version 5.1) software. The spectral ^1^H NMR region from δ = 0.56 to δ = 10.00 was segmented into regions with widths of 0.04 ppm, giving 232 integrated regions in each NMR spectrum.

### Cell culture

3 T3-L1 preadipocyte purchased from ATCC were cultured in 24 well plate at a density of 3×10^4^ cells/well. In DMEM containing 10% FBS, 2 mM glutamine, 20 mM Hepes, 50U/ml penicillin, and 50 mg/ml streptomycin sulfate. After 100% confluency, cells were cultured with differentiation medium (DMEM with 10% FBS, 0.5 mM IBMX, 2 mM DEX and 1.7 mM INS). After 48 h of stimulation, cells were cultured in DMEM supplemented with 10% FBS with/without PGBE and changed every two days up to 8 days.

### Oil Red O staining

Lipid accumulation of PGBE was examined by measuring triglyceride content using Oil-Red O staining. For Oil Red O staining, cells were washed gently with PBS twice, fixed with 3.7% fresh formaldehyde in PBS for 1 h at room temperature and stained with filtered Oil Red O solution (60% isopropanol and 40% water) for at least 1 h. After staining of lipid droplets with Red, the Oil Red O staining solution was removed and the plates were rinsed with water and dried. Images were collected on an Olympus microscope (Tokyo, Japan). Finally, the dye retained in the cells was eluted with isopropanol and quantified by measuring the optical absorbance at 500 nm.

### Multivariate statistics analysis

Principal component analysis (PCA) was performed using mean Pareto-scaled data obtained from aqueous solvent system. Then, partial least squares-discriminant analysis (PLS-DA) was also performed, which can yield a clearer differentiation of each class and enable a less complicated investigation of marker compounds

### Statistical analysis

Unless otherwise specified, all data are expressed as the mean ± standard error (SE) from triplicate experiments. One-way ANOVA (Scheffe test or student t test) was used for multiple comparisons using the Statistical Package for the Social Sciences (SPSS) program (version 16.0) (SPSS, Inc., Chicago, IL, USA). Values of p < 0.05 were considered statistically significant.

## Results and discussion

**Figure 1:**
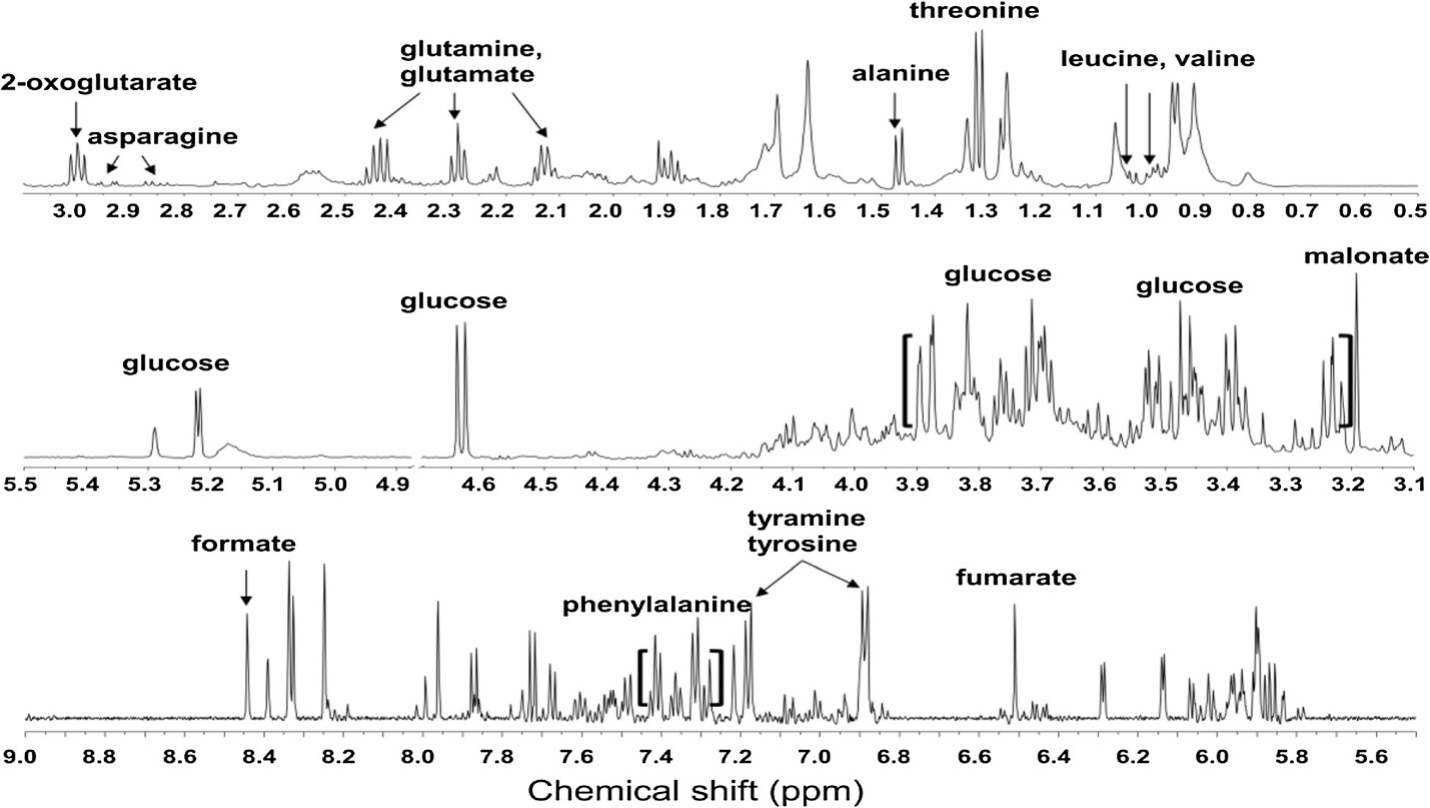
**Representative total** ^**1**^ **H NMR spectra (600 MHz) of the P. ginseng berry extract analyzed with direct D**_**2**_ **O extraction.**

Figure 1 shows a representative NMR spectrum of the D_2_O extracts from the PGBE at the mature stage. As described in Table 1, the signals were assigned based on comparisons with the database of the Chenomx NMR software suite: amino acid such as leucine, valine, threonine, alanine, glutamate, glutamine, asparagines, malonate, tyramine, tyrosine, and phenylalanine; organic acid such as 2-oxoglutarate, fumarate, and formate; sugars such as glucose.

**Table 1:** **Assignment of** ^**1**^ **H NMR spectral peaks obtained from P. ginseng berry extract analyzed by using D**_**2**_ **O solvent. S: singlet, d: doublet, t: triplet, m: multiplet, dd: doublet of doublet**

No	Compounds	Chemical shift (δ), Peak multiplicity, J value (Hz)
1	Leucine	0.95 (d, J = 6.0)
2	Valine	0.99 (d, J = 5.4), 1.03 (d, J = 7.1)
3	Threonine	1.32(d, J = 6.9)
4	Alanine	1.47 (d, J = 7.3)
5	Glutamate	2.10-2.16(m), 2.29 (t, J = 7.4, 2.48-2.48 (m)
6	Glutamine	2.10-2.16 (m), 2.40-2.48 (m)
7	Asparagine	2.85 (dd, J = 16.9, 7.7), 2.94 (dd, J = 16.9, 4.2)
8	2-Oxoglutaric acid	3.00 (t, J = 7.2)
9	Malonate	3.19 (s)
10	Glucose	3.21-3.25(m), 3.36-3.43 (m), 3.43-3.54 (m), 3.67-3.82 (m), 3.89 (dd, J = 11.9, 1.6), 4.63 (d, J = 8.0), 5.22 (d, J = 3.8)
11	Fumarate	6.51 (s)
12	Tyramine,	6.89 (d, J = 8.5), 7.18 (d, J = 8.3)
13	Tyrosine	6.89 (d, J = 8.5), 7.18 (d, J = 8.3)
14	Phenylalanine	7.31 (d, J = 7.8), 7.36 (t, J = 7.3), 7.41 (t, J = 7.6)
15	Formate	8.44 (s)

**Figure 2:**
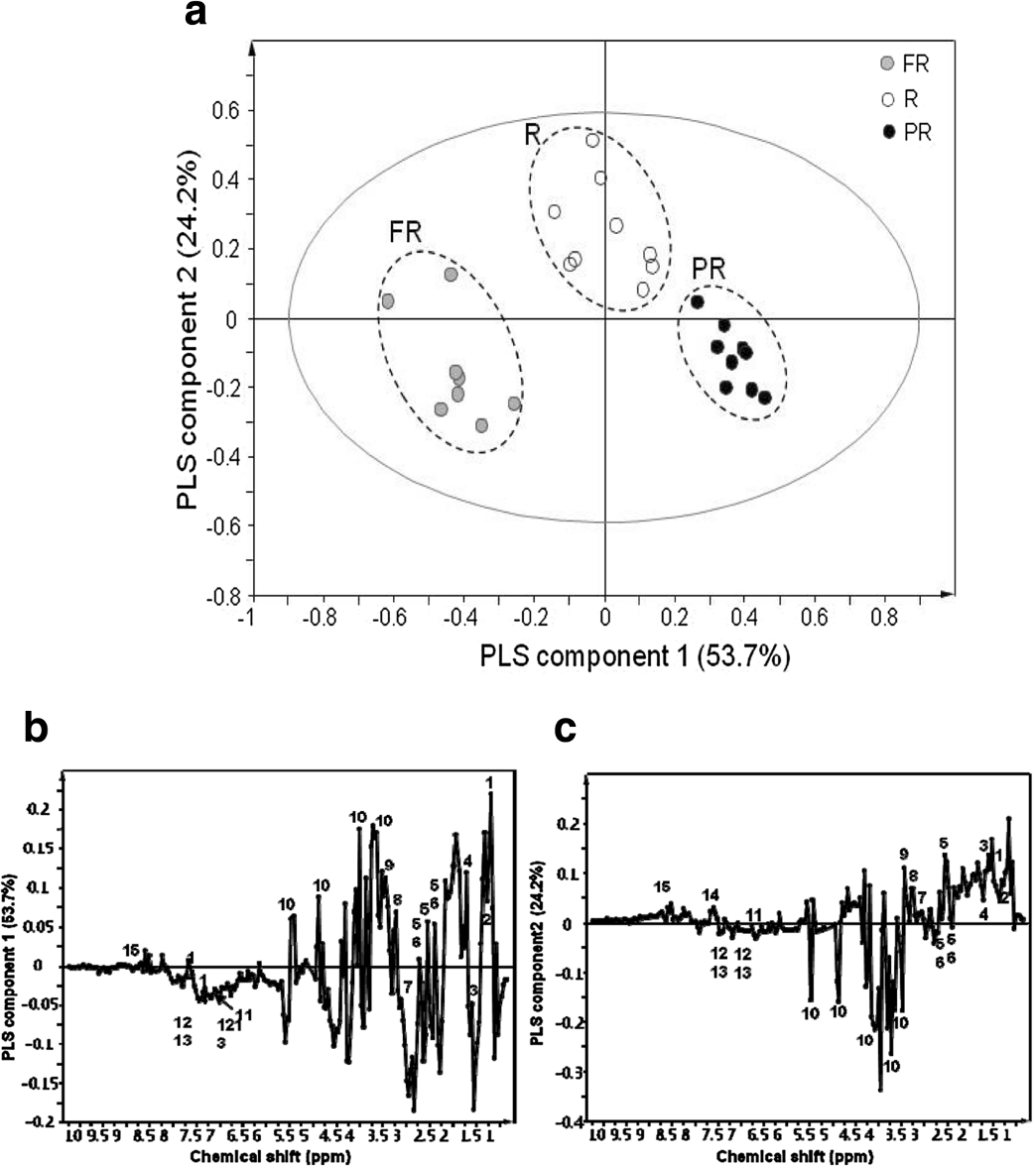
**PLS-DA-derived score plot (a) and loading plot (PLS component 1: b and PLS component 2: c) of P. ginseng berry extract analyzed with D**_**2**_ **O direct extraction.** (green circle symbol): PR, (blue circle symbol): R, (red circle symbol): FR . 1: leucine, 2:valine, 3: threonine, 4: alanine, 5: glutamate, 6: glutamine, 7: asparagines, 8: 2-oxoglutaric acid, 9: malonate, 10: glucose, 11: fumarate, 12: tyramine, 13: tyrosine, 14: phenylalanine, 15: formate.

PCA could clearly separate the maturation of ginseng berries based on the score plots (data not shown). PLS-DA was applied to the ^1^H NMR spectral data of P. ginseng berries according to three different maturation periods. The different maturation of the ginseng fruit samples were clearly distinguished based upon the PLS-DA-derived score plots (Figure 2a). Depending on the maturation of the sample, the positive direction was shifted to the negative direction of PC1. Loading plot analysis was performed to investigate the contributing metabolites for separating each PGBE from the score plots. As shown in Figure 2b, the levels of leucine, valine, alanine, glutamate, glutamine, 2-oxoglutarate, malonate, and glucose were higher in the PR stage samples than the FR berries. In addition, threonin, asparagines, fumarate, tyramine, tyrosine, and phenylalanine were higher in the FR berries than the PR ones. The levels of leucine, valine, threonin, alanine, asparagines, 2-oxoglutarate, malonate, phenylalanine, and formate were higher in the ripening fruits (Figure 2c) than other stages.

**Figure 3:**
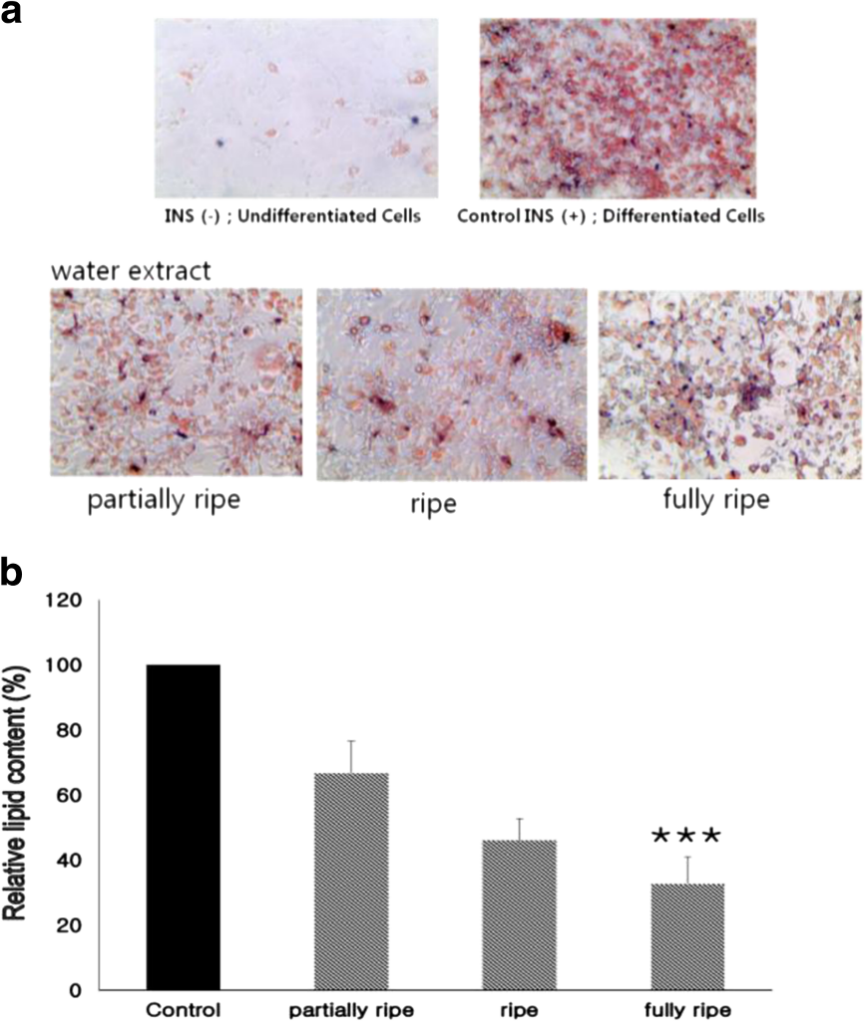
**Effects P. ginseng berry extract on Oil Red O staining in cultured 3 T3-L1 adipocytes.** 3 T3-L1 cells were treated with 100 μg/ml of P. ginseng berry extract. **(a)** Effects of P. ginseng berry extract on fat droplet formation in 3 T3-L1 cells. It was stained with Oil Red O dye and examined with a light microscope; **(b)** Relative lipid content by quantification method of Oil Red O staining. Data are presented as average ± SD (n =5). * indicates *** p <0.001 compared with control (expressed by t-test).

3 T3-L1 cells of the adipocyte morphology increase the synthesis and accumulation of triglycerides and acquire the signet ring appearance of adipose cells [14]. 3 T3-L1 cells are extensively used to study adipogenesis [15]. For this reason, we chose these cells for research to identity the feasibility of PGBE as a possible new anti-obesity herbal agent. This observation was further supported with the quantitative analysis of neutral lipid content by measuring the absorbance at 500 nm. 3 T3-L1 cells were treated with various maturation stage of PGBE. After 8 days, intracellular lipid accumulation was examined with Oil red O staining for lipid droplets as an indicator of the degree of adipogenesis. Figure 3a showed that the cell size was bigger and the intracellular fat drops were comparatively more in 3 T3-L1 cells. And comparatively adipogenesis and cell viability were decreased 100ug/ml treated group. In addition, the lipid accumulation rate was significantly reduced with PGBE treatment compared with the control; the decrease was maturation stage dependent (p <0.001); accumulation was about 67%, 46%, and 33% at PR, R, and FR by aqueous extraction concentration in Figure 3b.

In previous, many studies have reported the anti-obesity effects in various medicinal plants, such as Nigella sativa [16], Camellia sinensis [17], Hibiscus sabdariffa [18], Psyllium fibre [19], and Lycium barbarum [20]. Dey et al. [21] demonstrated the anti-obesity effect in Asian ginseng berry extract, and Attele et al. [22] also showed the anti-hyperglycemic effect in ginsenoside Re. According to Kim et al. [23], the group of people whom the black soybean peptide had been taken showed the decreased body mass and fat. The scientific study shows that natural products contain a large variety of components that possess lipid inhibition activity. Especially, a variety of herbs from plants have been used as traditional natural medicines for cure many kinds of diseases or restore to health. In particular, various oriental medicinal herbs are reported to have biological activity [24, 25].

In this study, it was confirmed that the amount of phenylalanine is higher in FR stage of P. ginseng berries, thus expecting to lower the obesity. Further in vivo research and clinical trials are still need to clarify the efficacy, safety, and precise molecular mechanisms of the anti-obesity effects of PGBE.

## Conclusion

In conclusion, this is the first study regarding metabolic profiling of PGBE using D_2_O solvent. Moreover, multiparameter pattern recognition analysis established ^1^H NMR spectra of PGBE. And PGBE efficiently inhibits adipogenesis in 3 T3-L1 adipocytes as indicated by significant reduction lipid accumulation. It is predicted that P. ginseng berry extract may apparently inhibit the adipogenic differentiation and lipid accumulation in the cells through the activation of various adipogenic regulatory genes such as peroxisome proliferator-activated receptor (PPARγ) and CCAAT element binding protein (C/EBP-α). Further the mechanism underlying the anti-adipogenic activity of P. ginseng berry extract has to be studied in the future.
